# Development of a personalized cellular ex-vivo cbl-b silencing cancer immunotherapy

**DOI:** 10.1186/2051-1426-2-S3-P223

**Published:** 2014-11-06

**Authors:** Guenther Lametschwandtner, Hans Loibner, Monika Sachet, Hubert Hayden, Michaela Hassler, Manfred Schuster, Marc Salzberg, Pierre Triozzi, Josef Friedl

**Affiliations:** 1 Apeiron Biologics AG, Vienna, Austria; 2 Department of Surgery, General Hospital of Vienna, Austria; 3 Wake Forest University, Winston-Salem, NC, USA

## Background

The E3 ubiquitin ligase cbl-b has been identified as an important gatekeeper limiting T cell activation. Concordantly, the immune system of cbl-b deficient mice can effectively fight tumors, thereby validating cbl-b as an excellent target to enhance anti-tumor immune activity. We have recently shown in proof-of-concept experiments that transfer of transiently cbl-b silenced murine T cells had efficacy to enhance the anti-tumor immune response in mouse models.

## Methods

A design algorithm was used to screen for siRNAs that are highly effective to silence cbl-b, and the optimized siRNA was produced at a GMP manufacturer. PBMCs were isolated from healthy donors or cancer patients, transfected with siRNA by electroporation and immune cell phenotype and activation was determined by FACS and ELISA. For enhancement of DC vaccination responses, PBMCs were ex vivo silenced, and co-administrated with the DC preparations intranodally to the cancer patient.

## Results

We have established a highly efficient transfection protocol using a commercial electroporation device enabling us to simultaneously transfect T, B, NK cells and monocytes with minimal cell damage. Using this protocol, we have identified a siRNA that was able to shut down cbl-b expression for more than 7 days in stimulated human T cells, resulting in strong enhancement of T cell activation, cytokine production and proliferation. Moreover, simultaneous silencing of cbl-b in all immune cells of the PBMCs yielded additional advantages, most notably enhancing NK cell reactivity against tumor cell and IL-2 stimulation. Silencing of cancer patient PBMCs yielded similar results ex vivo and intranodal transfer of autologous cbl-b silenced cells together with activated DCs to patients with advanced cancers was feasible and well tolerated.

## Conclusions

To enable the clinical implementation of a cbl-b ex vivo silencing treatment, we have established and tested a protocol that can be easily performed on any clinical unit that applies adoptive cell therapies to patients. Based on these results, a Phase I trial for the systemic administration of cbl-b silenced PBMCs to patients with advanced cancers is presently being set up.

